# The association between narcolepsy during pregnancy and maternal-fetal risk factors/outcomes

**DOI:** 10.5935/1984-0063.20220054

**Published:** 2022

**Authors:** Annise Wilson, Deepa Dongarwar, Krystal Carter, Maricarmen Marroquin, Hamisu M Salihu

**Affiliations:** 1 Baylor College of Medicine, Center of Excellence in Health Equity, Training, and Research - Houston - TX - United States; 2 Baylor College of Medicine, Department of Family Medicine - Houston - TX - United States

**Keywords:** Narcolepsy, Cataplexy, Pregnancy, Delivery, Obstetric, Morbidity, Hospitalization

## Abstract

**Objective:**

We sought to determine whether narcolepsy in pregnancy is associated with adverse maternal-fetal outcomes.

**Material and Methods:**

A retrospective, cross-sectional analysis was performed using the nationwide inpatient sample (NIS) for the period 2008-2017. The primary exposure was narcolepsy with cataplexy, narcolepsy type 1 (NT1), and without cataplexy, narcolepsy type 2 (NT2), and the endpoints were a composite of maternal-fetal outcomes or risk factors.

**Results:**

A total of 7,742 hospitalizations among pregnant women with narcolepsy were identified (prevalence = 17.6 per 100,000), of which 6,769 (88%) were diagnosed with NT2. Statistically significant positive associations were found between narcolepsy and the following conditions: obesity (odds ratio (OR): 2.99, confidence interval (CI): 2.4-3.74), anemia (OR=1.41, CI: 1.13-1.77), pre-pregnancy hypertension (OR=1.93, CI: 1.37-2.7), pre-pregnancy diabetes (OR=1.7, CI: 1.08-2.84), and gestational hypertension (OR=1.58, CI: 1.13-2.20) in the ICD-9 group. Similar findings were noted in the ICD-10 group with the exception of gestational hypertension, gestational diabetes, and anemia.

**Conclusion:**

Given these important findings, we propose a global approach of screening for narcolepsy among women of reproductive age with pre-existing risk factors prior to conception to minimize pregnancy complications.

## INTRODUCTION

Narcolepsy is a chronic hypersomnia that is separated into two types and formally named type 1 and type 2. Following the International Classification of Sleep Disorders, third edition (ICSD-3), type 1 narcolepsy can be characterized by cataplexy among other findings including a low CSF-hypocretin-1 concentration while type 2 narcolepsy is not associated with cataplexy^1^. The prevalence of type 1 narcolepsy (NT1) is approximately 0.05% in the United States, with onset between 15 and 35 years of age^2^,^3^. The true prevalence of type 2 narcolepsy (NT2) is not known as the presentation is more variable though it is estimated to be higher than that of type 1. It is estimated that only 25% of people who have narcolepsy have been diagnosed and are receiving treatment. There are no gender differences in the rates of narcolepsy^3^.

Symptoms of narcolepsy include excessive daytime sleepiness (with sleep attacks), sleep paralysis, hypnagogic and hypnopompic hallucinations, and REM behavior disorder. Cataplexy is a sudden loss of muscle tone that is provoked by experiencing a typically strong positive emotion, such as laughter. This occurs due to the intrusion of REM atonia into the wake state^2^. Comorbidities include type 2 diabetes mellitus and obstructive sleep apnea. Of note, weight gain is prevalent in individuals with narcolepsy, with an estimated 30% of patients fitting this description^2^. It is hypothesized that the underlying cause for the weight gain may be a lack of orexin/hypocretin, which leads to decreased metabolism along with decreased appetite, though to a lesser degree^4^,^5^.

Approximately 95% of individuals with NT1 have a deficiency of hypocretin (orexin)-producing neurons in the lateral hypothalamus^6^. Orexin A and orexin B (also known as hypocretin 1 and hypocretin 2, respectively) are neuropeptides that regulate arousal, wakefulness, and appetite. In humans, the orexin A level is severely reduced or undetectable in the cerebrospinal fluid (CSF) of approximately 90% of patients with NT1. NT1 is characterized by a low orexin A level (<110pg/ml) and cataplexy^7^.

Multiple studies have suggested differences in prevalence among racial and ethnic groups^6^,^8^-^10^. This difference is thought to arise from human leukocyte antigen (HLA) types as narcolepsy is tightly associated with HLA-DR2, HLA-DQA1, and HLA-DQB1*0602^8^,^9^,^11^. HLA-DQB1*0602 has been found to be more prevalent in individuals with cataplexy^12^. The HLA-DQB1*06:02 allele is strongly associated with narcolepsy and is present in over 98% of individuals with narcolepsy type 1 and about 50% of individuals with narcolepsy type 2^13^. Prior studies have suggested that African Americans are more likely to be HLA DQB1*0602 positive and hypocretin deficient when compared to Caucasians, Latinos, and Asians^9^,^10^.

Maternal-fetal outcomes have been studied extensively in obstructive sleep apnea^14^,^15^, but studies on narcolepsy are lacking. Prior studies have included retrospective case-control and cohort designs. Research questions included whether caesarean sections in pregnant women with cataplexy was indicated as well as the appropriate management of narcolepsy during pregnancy and lactation^16^. A European study found that less than 1% of pregnant women with cataplexy experienced cataplexy during delivery^17^. The same cohort study found that weight gain during pregnancy was higher in women with narcolepsy as well as the rate of impaired glucose metabolism^17^. The mean birth weight appeared to be within a normal range as was the gestational age. However, another study found higher rates of gestational diabetes. The aim of this paper is to provide updated information on the impact of narcolepsy on maternal-fetal outcomes using a nationally representative dataset covering the entire United States (US).

## MATERIAL AND METHODS

We conducted a cross-sectional analysis of hospitalization records from January 1, 2008 through December 31, 2017 using the Nationwide Inpatient Sample (NIS)^18^. The NIS datasets constitute the largest all-payer, publicly available inpatient database in the US and are made available by the Healthcare Cost and Utilization Project (HCUP). The systematic sampling strategy ensures that hospitalizations in the NIS are representative of the population on important factors including month of admission, primary reason for hospitalization, and hospital size, location, ownership, and teaching status; and the result is an approximate 20% sample of hospital discharges from participating states, totaling seven million inpatient hospitalizations each year (35 million when weighted) from the 47 participating states.

Our study sample included pregnancy hospitalizations among women within the age range of 18 to 40 years. Diagnoses and procedures were coded using International Classification of Diseases, Ninth Revision, Clinical Modification (ICD-9-CM) diagnosis codes until the 3^rd^ quarter of 2015, after which HCUP transitioned to ICD-10-CM format. To assess the study’s primary exposure, we first scanned the up to 30 diagnosis codes in each patient’s discharge record for an indication of narcolepsy with or without cataplexy. We next sub-divided these encounters into two mutually exclusive groups: 1) narcolepsy with cataplexy; and 2) narcolepsy without cataplexy. Neither the timing of the narcolepsy diagnosis nor the medication status during pregnancy was listed in the NIS dataset. The maternal outcomes/risk factors for the study included obesity, anemia, pre-pregnancy and gestational diabetes, pre-pregnancy, and gestational hypertension, preeclampsia and eclampsia. The delivery outcomes for the study were C-section, early delivery and stillbirth. Table 1 shows the list of ICD-9-CM and ICD-10-CM codes utilized for identifying the exposure and outcome variables for this study. We created a composite variable ‘any risk factor’, based on presence of any of the adverse maternal or delivery outcomes mentioned above.

**Table 1:** ICD-9 and ICD-10 codes utilized for the exposure and outcome variables.

Condition	International Classification of Diseases, 9^th^ Edition, Diagnosis Code•	International Classification of Diseases, 10^th^ Edition, Diagnosis Code•
**Exposure**		
Narcolepsy with or without cataplexy	347.x	G47.4x
Narcolepsy with cataplexy	347.01,347.11	G47.411,G47.421
Narcolepsy without cataplexy	347.00,347.10	G47.419,G47.429
**Maternal outcomes**		
Gestational diabetes	648.0x, 648.8x	O24.4x, O24.9x, O99.81x
Preeclampsia	642.4x, 642.5x	O14.x
Eclampsia	642.6x	O15.x
Gestational hypertension	642.3x	O13.x
Obesity	278.00, 278.01 , 278.03, 649.1x, V85.3x, V85.4x, 793.91	E66.0x, E66.1, E66.2, E66.8, E66.9, Z68.3x,Z68.4x,R93.9
Anemia	280x, 281x, 282x, 283x, 284x, 285x, 648.2x	D5x, D60x,D61x, D62x, D63x, D64x,O99.0x
Pre-pregnancy hypertension	401x, 402x, 403x, 404x, 405x, 642.0x, 642.1x, 642.2x, 642.7x	I10x,I11x,I12x,I13x,I15x,I16x,O10x,O11x, O16x
Pre-pregnancy diabetes	249x, 250x, 648.0x	E08x, E09x, E10x, E11x, E13x, O24.0x,O24.1x,O24.3x,O24.8x
**Delivery outcomes**		
Cesarean section	669.7x	O82
Early-onset delivery	644.2x	O60.x
Stillbirth	656.4x, V27.1, V27.3 , V27.4, V27.6 , V27.7	O34.4x, Z37 .1, Z37.3 , Z37.4, Z37.6 , Z37.7

Note: The code suffix "x" represents all possible codes that follow the stated code prefix.

For each inpatient hospitalization, the NIS database captures various sociodemographic, clinical, and hospital characteristics. Patient age in years was categorized as 18-24, 25-29, 30-34, and 35-40. Self-reported race/ethnicity, which is reported differently across states, was standardized by first grouping this as Hispanic or non-Hispanic (NH), and then further classifying the non-Hispanics by race (NH-White, NH-Black, or other). Insurance status was based on the primary payer for the hospitalization, and was classified into Medicare, Medicaid, private, self-pay and other. Socioeconomic status was estimated from the median household income in the patient’s zip code of residence, and estimated values were classified into quartiles. Hospital characteristics captured included: US census region (Northeast, Midwest, South, and West), hospital size based on the number of short-term acute beds in a hospital (small, medium, and large), and location/teaching status (urban-teaching, urban-non-teaching, and rural).

We conducted joinpoint regression analyses to evaluate and describe the trends in rates of narcolepsy with/without cataplexy, narcolepsy with cataplexy, and narcolepsy without cataplexy over the study period 2008-2017. Joinpoint regression is a statistical modeling approach specifically designed to evaluate and describe the extent to which the rate of an outcome changes over time. The procedure first fits the annual rates of the outcome of interest to a model with the minimum number of joinjoints (zero), suggesting that a straight line and single trend best fits the annual prevalence data^19^. Then, more joinpoints are added iteratively to test the statistical significance of the various models using Monte Carlo permutation method^19^. Once the final (best-fitting) model with the optimal number of joinpoints has been selected, the overall trend over the study period is characterized using average annual percent change (AAPC) measure and its 95% confidence interval (CI).

We conducted bivariate analyses to compare the socio-demographic and hospital characteristics across pregnant women grouped as having narcolepsy with/without cataplexy, narcolepsy with cataplexy and narcolepsy without cataplexy. Descriptive statistics were utilized to derive the prevalence of each of the maternal and delivery outcomes among the three exposure groups. Lastly, we conducted unadjusted and adjusted survey logistic regression model to assess the association between narcolepsy with/without cataplexy and each of the maternal and delivery outcomes. We conducted sensitivity analysis to evaluate the association between our exposure and outcome for the entire study period and for 2008-2015, 3^rd^ quarter time period. This was done to study the impact of change from ICD-9-CM to ICD-10-CM format from the 4^th^ quarter of 2015. All statistical analyses for the study were performed using R (version 3∙6∙1) and RStudio (version 1.2.5001) and the trends analyses were run using Joinpoint Regression Program, version 4.7.0.0 (National Cancer Institute). We assumed a 5% type I error rate for all hypothesis tests. This study was deemed exempt by the IRB of Baylor College of Medicine as the study was performed on publicly available, de-identified data.

## RESULTS

We analyzed a total of 43,797,082 pregnancy hospitalizations, of which 7,702 had a diagnosis of narcolepsy (prevalence = 17.6 per 100,000). The prevalence of NT1 and NT2 was 2.1 per 100,000 and 15.5 per 100,000, respectively; with NT2 accounting for most of the cases of narcolepsy (88%). Table 2 portrays the distribution of all narcolepsy, NT1 and NT2 by maternal sociodemographic features, discharge status, and hospital characteristics. Exclusive of mothers with missing information about age (about 2.8% of them), the prevalence of all narcolepsy, NT1 and NT2 increased progressively with maternal age reaching a zenith among oldest mothers (30-40 years). Of the available information provided, the overwhelming majority of cases of narcolepsy was accounted for by NH-Whites (71.1%) who also had the highest prevalence of narcolepsy regardless of the subtype. NH-Blacks followed with the second highest prevalence.

**Table 2:** Sociodemographics of pregnant women with narcolepsy with or without cataplexy.

Total pregnancy hospitalizations		Narcolepsy with or without cataplexy			Narcolepsy with cataplexy (NT1)			Narcolepsy without cataplexy (NT2)		
		n=7702	%=100	Prevalence per 100,000 hospitalizations	n=938	%=100	Prevalence per 100,000 hospitalizations	n=6769	%=100	Prevalence per 100,000 hospitalizations
*Age*										
18-24 years	12697220	1201	15.6%	9.5	224	23.9%	1.8	976	14.4%	7.7
25-29 years	12664112	1870	24.3%	14.8	263	28.0%	2.1	1608	23.8%	12.7
30-34 years	11559164	2447	31.8%	21.2	237	25.3%	2.1	2210	32.6%	19.1
35-40 years	6258439	1928	25.0%	30.8	199	21.2%	3.2	1734	25.6%	27.7
Missing	618147	256	3.3%	41.4	15	1.6%	2.4	241	3.6%	39.0
*Race/Ethnicity*										
NH-White	21129103	5474	71.1%	25.9	662	70.6%	3.1	4812	71.1%	22.8
NH-Black	6305449	1077	14.0%	17.1	94	10.0%	1.5	988	14.6%	15.7
Hispanic	8248856	282	3.7%	3.4	49	5.2%	0.6	233	3.4%	2.8
Other	4235134	242	3.1%	5.7	25	2.7%	0.6	217	3.2%	5.1
Missing	3878539	628	8.2%	16.2	109	11.6%	2.8	519	7.7%	13.4
*Disposition*										
Routine	42170008	7010	91.0%	16.6	839	89.4%	2.0	6176	91.2%	14.6
Transfer	434653	314	4.1%	72.2	50	5.3%	11.5	264	3.9%	60.7
Discharged AMA	245118	70	0.9%	28.6	-	-	-	65	1.0%	26.5
Other	904762	298	3.9%	32.9	44	4.7%	4.9	254	3.8%	28.1
Missing	15725	-	-	-	-	-	-	-	-	-
*Household Income*										
Lowest quartile	12348446	1881	24.4%	15.2	179	19.1%	1.4	1707	25.2%	13.8
Second quartile	10926465	2035	26.4%	18.6	247	26.3%	2.3	1787	26.4%	16.4
Third quartile	10576457	2037	26.4%	19.3	283	30.2%	2.7	1754	25.9%	16.6
Highest quartile	9243429	1695	22.0%	18.3	219	23.3%	2.4	1476	21.8%	16.0
Missing	702285	54	0.7%	7.7	-	-	-	44	0.7%	6.3
*Primary Payer*										
Medicare	2785764	1071	13.9%	38.4	168	17.9%	6.0	903	13.3%	32.4
Medicaid	13613792	783	10.2%	5.8	93	9.9%	0.7	690	10.2%	5.1
Private	15466812	1887	24.5%	12.2	232	24.7%	1.5	1655	24.4%	10.7
Other	1923034	186	2.4%	9.7	-	-	-	176	2.6%	9.2
Missing	10007679	3775	49.0%	37.7	435	46.4%	4.3	3345	49.4%	33.4
*Hospital Region*										
Northwest	7150793	1116	14.5%	15.6	158	16.8%	2.2	959	14.2%	13.4
Midwest	9350291	2586	33.6%	27.7	339	36.1%	3.6	2247	33.2%	24.0
South	16855552	2931	38.1%	17.4	313	33.4%	1.9	2623	38.8%	15.6
West	10440444	1069	13.9%	10.2	129	13.8%	1.2	940	13.9%	9.0
*Hospital Bed Size*										
Small	5995364	1086	14.1%	18.1	110	11.7%	1.8	981	14.5%	16.4
Medium	12431079	2007	26.1%	16.1	257	27.4%	2.1	1750	25.9%	14.1
Large	25156287	4589	59.6%	18.2	571	60.9%	2.3	4018	59.4%	16.0
Missing	214350	20	0.3%	9.3	-	-	-	20	0.3%	9.3
*Hospital Location and Teaching Status*										
Rural	4412453	631	8.2%	14.3	73	7.8%	1.7	558	8.2%	12.6
Urban non-teaching	14439394	2019	26.2%	14.0	178	19.0%	1.2	1841	27.2%	12.7
Urban teaching	24730883	5032	65.3%	20.3	687	73.2%	2.8	4350	64.3%	17.6
Missing	214350	20	0.3%	9.3	-	-	-	20	0.3%	9.3

Note: Prevalence represents the rate of outcomes (narcolepsy with or without cataplexy, narcolepsy with cataplexy, narcolepsy without cataplexy) in each of the patient characteristic groups. As per HCUP guidelines, values less than or equal to 10 are suppressed to prevent patient identification.

More than 90% of hospitalized mothers diagnosed with narcolepsy were routinely discharged although the prevalence of narcolepsy was highest among those transferred to other facilities. Among those with available information on income, mothers in the lowest household income bracket appeared to have the least prevalence of narcolepsy; however, there was only minimal variation across the remaining income groups. While patients covered by Medicare had the highest prevalence of narcolepsy, those on Medicaid had the lowest. Narcolepsy prevalence was also greatest in the Midwest but lowest in the West. Most of the diagnosed cases of narcolepsy among pregnant women were documented in medium and large hospitals (accounting for >85% of cases), and in urban non-teaching and teaching hospitals.

Table 3 summarizes the frequencies of the maternal-fetal outcomes in pregnant women with narcolepsy. There is a noticeable increase in the rates of obesity as well as pre-pregnancy hypertension in both NT1 and NT2 narcolepsy groups (17.4% and 18.7% of our respective population of interest displaying obesity compared to 7.2% in the general population; and 8% and 13.9% of the same population displaying hypertension compared to 4.1% in the general population). The percentages of anemia and pre-pregnancy diabetes also increased in the narcolepsy without cataplexy group (for anemia this percentage jumps to 16.5% compared to 13.7% in the general population and for pre-pregnancy diabetes the percentage is 6.1% compared to 2.3% in the general population. Gestational hypertension and pre-eclampsia were more common in the narcolepsy with cataplexy group (for gestational hypertension the percentage increases to 5.3% compared to 3.5% in the general population and for pre-eclampsia the percentage increases to 4.8% compared to 3.6% in the general population). Caesarean sections were also more common in the narcolepsy with cataplexy group (1.1% compared to 0.2% in the general population). Unlike the previous condition, the rates of gestational diabetes, eclampsia, and preterm delivery were not increased in pregnant women with narcolepsy.

**Table 3:** Frequencies of various pregnancy and delivery outcomes/risk factors among women with all narcolepsy (regardless of subtype).

Outcomes	Narcolepsy with or without cataplexy			Narcolepsy with cataplexy			Narcolepsy without cataplexy		
	**No=43785396**	**Yes=7702**	**Prevalence per 10,000 hospitalizations**	**No=43792160**	**Yes=938**	**Prevalence per 10,000 hospitalizations**	**No=43786329**	**Yes=6739**	**Prevalence per 10,000 hospitalizations**
**Maternal characteristics**	**n**	**n**		**n**	**n**		**n**	**n**	
**Obesity**									
No	40640526	6279	1.54	40646030	775	0.19	40641301	5504	1.4
Yes	3144870	1423	4.52	3146130	163	0.52	3145028	1265	4.0
**Anemia**									
No	37772346	6453	1.71	37777994	805	0.21	37773146	5653	1.5
Yes	6013050	1250	2.08	6014166	133	0.22	6013183	1117	1.9
**Pre-pregnancy hypertension**									
No	41992008	6690	1.59	41997835	863	0.21	41992872	5827	1.4
Yes	1793388	1012	5.64	1794325	75	0.42	1793458	942	5.2
**Pre-pregnancy diabetes**									
No	42781446	7263	1.70	42787796	913	0.21	42782355	6355	1.5
Yes	1003950	439	4.37	1004364	25	0.25	1003975	414	4.1
**Gestational hypertension**									
No	42261182	7414	1.75	42267708	888	0.21	42262066	6531	1.5
Yes	1524214	288	1.89	1524452	50	0.33	1524264	238	1.6
**Gestational diabetes**									
No	40833992	7311	1.79	40840419	884	0.22	40834870	6432	1.6
Yes	2951404	392	1.33	2951741	55	0.19	2951459	337	1.1
**Preeclampsia**									
No	42223849	7456	1.77	42230412	893	0.21	42224738	6567	1.6
Yes	1561547	247	1.58	1561748	45	0.29	1561592	202	1.3
**Eclampsia**									
No	43746495	7693	1.76	43753249	938	0.21	43747428	6760	1.5
Yes	38901	-	-	38911	-	-	38901	-	-
**Delivery characteristics**									
**C-section**									
No	43703256	7683	1.76	43710010	928	0.21	43704179	6760	1.5
Yes	82140	19	2.31	82150	-	-	82150	-	-
**Early delivery**									
No	41419196	7427	1.79	41425735	888	0.21	41420080	6543	1.6
Yes	2366200	276	1.17	2366425	50	0.21	2366250	226	1.0
**Stillbirth**									
No	43528596	7687	1.77	43535345	938	0.22	43529529	6754	1.6
Yes	256800	15	0.58	256815	-	-	256800	15	0.6
**Composite outcome**									
**Any risk factor**									
No	28415179	4083	1.44	28418727	535	0.19	28415715	3548	1.2
Yes	15370217	3619	2.35	15373433	403	0.26	15370615	3221	2.1

Note: As per HCUP guidelines, values less than or equal to 10 are suppressed to prevent patient identification.

**Figure 1:**
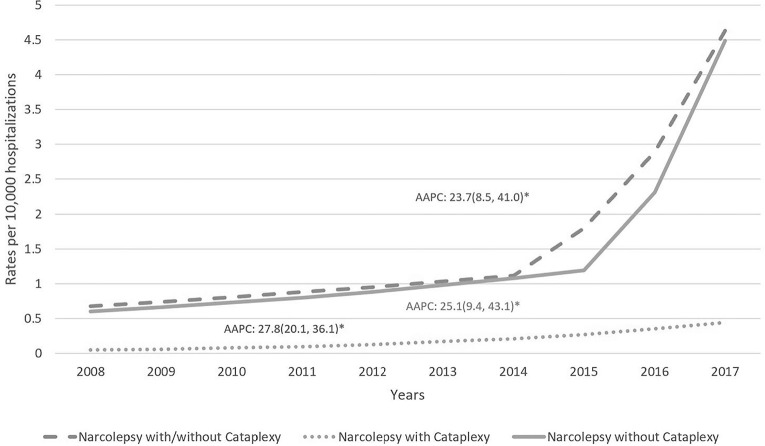
Rates of narcolepsy, (NT1 and NT2) per 10,000 pregnancy hospitalizations in the US, 2008-2017.

Due to coding differences related to the transition from ICD-9 to ICD-10 (shown in Figure 1) in 2015, the results have been split to distinguish these periods. Overall there was a 27.8% average annual increase in the rates of narcolepsy hospitalizations over the study period (AAPC: 27.8, 95%CI: 20.1, 36.1). Table 4 lists the unadjusted and adjusted odds ratios for the association between all narcolepsy (NT1 and NT2) and various outcomes or risk factors. Statistically significant findings among pregnant women with narcolepsy (regardless of subtype) on the aforementioned maternal-fetal outcomes/risk factors include obesity (AOR=2.99, 95%CI: 2.40-3.74), anemia (AOR=1.41, 95%CI: 1.13-1.77), pre-pregnancy hypertension (AOR=1.93, 95%CI: 1.37-2.7), pre-pregnancy diabetes (AOR=1.7, 95%CI: 1.08-2.84), and gestational hypertension (AOR=1.58, 95%CI: 1.13-2.20) in the ICD-9 era. In the overall study period, gestational hypertension, gestational diabetes and anemia were not found to have a statistical association with narcolepsy. Similarly, caesarean section, pre-term delivery or stillbirth were not associated with narcolepsy in pregnant women.

**Table 4:** Unadjusted and adjusted survey logistic regression models to assess the association between narcolepsy with/without cataplexy and various outcomes.

Outcomes	Narcolepsy/cataplexy (all years)		Narcolepsy/cataplexy (2008-2015 3^rd^ quarter)	
	Unadjusted OR	Adjusted OR	Unadjusted OR	Adjusted OR
**Maternal characteristics**				
Obesity	2.93(2.57-3.34)^*^	2.06(1.80-2.36)^*^	3.14(2.52-3.91)^*^	2.99(2.40-3.74)^*^
Anemia	1.22(1.06-1.40)^*^	1.11(0.97-1.28)	1.35(1.09-1.69)^*^	1.41(1.13-1.77)^*^
Pre-pregnancy hypertension	3.54(3.06-4.11)^*^	1.90(1.62-2.23)^*^	2.51(1.80-3.50)^*^	1.93(1.37-2.70)^*^
Pre-pregnancy diabetes	2.58(2.06-3.22)^*^	1.41(1.11-1.79)^*^	1.93(1.15-3.22)^*^	1.70(1.08-2.84)^*^
Gestational hypertension	1.08(0.82-1.41)	1.01(0.77-1.32)	1.75(1.25-2.45)^*^	1.58(1.13-2.20)^*^
Gestational diabetes	0.74(0.59-0.93)^*^	0.80(0.64-1.01)	1.24(0.95-1.63)	1.19(0.91-1.56)
Preeclampsia	0.89(0.66-1.21)	0.92(0.68-1.24)	1.41(0.96-2.07)	1.37(0.93-2.02)
Eclampsia	1.39(0.39-5.60)	1.35(0.34-5.43)	3.45(0.86-4.89)	3.50(0.87-5.06)
**Delivery outcomes**				
C-section	1.34(0.50-3.60)	1.51(0.57-4.04)	1.49(0.37-6.02)	1.51(0.37-6.08)
Early delivery	0.65(0.50-0.85)^*^	0.77(0.59-1.01)	1.18(0.87-1.59)	1.18(0.87-1.59)
Stillbirth	0.33(0.11-1.03)	0.35(0.11-1.07)	0.58(0.13-2.08)	0.51(0.13-2.06)
**Composite outcome**				
Any risk factor	1.64(1.48-1.81)^*^	1.38(1.25-1.53)^*^	1.68(1.44-1.97)^*^	1.65(1.41-1.93)^*^

Notes: OR = Odds ratio;

* Statistically significant; Models are adjusted for age, race, disposition, primary payer, household income, hospital region, hospital bed-size and hospital location, and teaching status.

## DISCUSSION

In this study, we observed significant positive associations between narcolepsy and the following risk factors and pregnancy-related conditions: maternal obesity, anemia, pre-pregnancy hypertension and diabetes, and gestational hypertension. These associations persisted after adjusting for potential confounders such as age, race, disposition, and income. The prevalence of narcolepsy with and without cataplexy in this study matches that of previously published data^3^. Similar to Calbo-Ferrandiz’s study^16^, there was not an increased rate of preterm labor or caesarean sections in the narcolepsy group. In addition, obesity was more prevalent in the narcolepsy group as was anemia and pre-pregnancy diabetes, findings that are consistent with those of other investigators^17^.

Our study also revealed that pregnant women with narcolepsy were older than those without narcolepsy. This may explain why there was a higher prevalence of obesity, as BMI tends to increase with age, though narcolepsy alone is associated with obesity.

Given the fact that non-Hispanic blacks are more likely to be HLA-DQB1*0602 gene positive^9^,^11^,^12^ , we expected a higher prevalence of narcolepsy but the rates were unchanged. This could be explained by under-diagnosis of narcolepsy in pregnant women or the attribution of clinical features of narcolepsy to other clinical conditions that tend to present with similar features (e.g., obstructive sleep apnea).

The possible overdiagnosis of sleep apnea may be explained by confounding factors such as the fact that African Americans are more likely to have a higher BMI than are their White counterparts^20^. Surprisingly, our results show the regional patterns in narcolepsy prevalence coincide with that of the regions with the highest BMI such as the South and the Midwest. Studies have shown an association between socioeconomic status and sleep quality^21^,^22^. We found that the narcolepsy diagnosis was lowest in those at the lowest income quartile and highest in those at the third income quartile.

To our knowledge, this is the largest study in the United States evaluating maternal-fetal risk factors/outcomes in pregnant women with narcolepsy. When coupled with the association that narcolepsy can occur simultaneously with obstructive sleep apnea in approximately 24% of cases^23^, appropriate screening prior to conception is essential in order to minimize adverse maternal-fetal outcomes. From the research, pregnant women typically are not often screened for narcolepsy due to the similarities that present in pregnancy, obstructive sleep apnea and narcolepsy. However, narcolepsy presents unique challenges that prevent treatment while pregnant as many of the available options are deemed to be potentially teratogenic, but overall there is inadequate data which leads to varied management by clinicians^24^,^25^. Many women with narcolepsy will discontinue pharmacotherapy during pregnancy and resort to alternative management strategies, though others will continue medications during pregnancy with stimulants and antidepressants most commonly used. Pascoe et al. (2019)^26^ found that when comparing the various pharmacotherapy groups used to treat narcolepsy, pregnancy and fetal outcomes were comparable.

One major limitation of the study is that the data incorporates both ICD-9 and ICD-10 data which are not comparable. Based on Figure 1, it appears that the prevalence of narcolepsy increased dramatically after 2015. However, this could be attributed in large part to the International Classification of Disease being updated from ICD-9 to ICD-10 in 2015. During this transition, ailments had to be diagnosed and recorded in more detail which may have led to errors in coding. Implementing this new system caused an increase in codes from 14,000 ICD-9 codes to over 70,000 ICD-10^27^. One could consider the increase of narcolepsy diagnosis due in part to increased clinician awareness stemming doctors having to spend more time writing the increased documentation needed for ICD-10. We hypothesize that the change in coding from ICD-9 to ICD-10 had a side effect of increasing the number of diagnosed cases for narcolepsy without there being a true increase in incidence. More research needs to be done on this topic to ascertain the exact cause for this change in prevalence. In addition, there may be a sample bias, missing data, and testing differences among hospitals in the National Information System. It is possible that some of these findings may be explained by medications taken during pregnancy, but this data was not included in the NIS. Pregnant women are more likely to suffer for sleep disturbances such as insomnia and obstructive sleep apnea^28^. Future studies should examine if the association between lifestyle factors and socio-economic status on narcolepsy in pregnant women, is similar to the one that has already been established in the general population^29^,^30^.

In conclusion, the findings of this study reveal significant differences in maternal-fetal outcomes/risk factors in pregnant women with narcolepsy. Given these important findings, we propose a global approach of screening for narcolepsy among women of reproductive age with pre-existing risk factors prior to conception to minimize adverse maternal-fetal outcomes.
